# A toolkit for generating virtual brightfield images of histological and immunohistochemical stains from multiplexed data with AI-based channel selection and image enhancement

**DOI:** 10.3389/fbinf.2026.1765143

**Published:** 2026-03-09

**Authors:** Tristan Whitmarsh, Mohammad Al Sa’d, Eduardo González Solares, Alireza Molaeinezhad, Melis O. Irfan, Claire Mulvey, Marta Paez-Ribes, Atefeh Fatemi, Wei Cope, Kui Hua, Gregory Hannon, Dario Bressan, Nicholas Walton

**Affiliations:** 1 Institute of Astronomy, University of Cambridge, Cambridge, United Kingdom; 2 CRUK Cambridge Institute, Li Ka Shing Centre, University of Cambridge, Cambridge, United Kingdom; 3 Cambridge University Hospitals NHS Foundation Trust, Cambridge, United Kingdom

**Keywords:** computational histology, deep learning, digital pathology, H&E, IHC, LLM, multiplex imaging, virtual staining

## Abstract

Multiplex imaging provides valuable insights into the functional and spatial organization of cells and tissues. However, traditional brightfield histopathology imaging remains important and may be required alongside multiplex imaging. We introduce a generalized framework to generate virtual brightfield images from multiplexed data, thereby reducing the need for additional tissue preparation and alignment with the multiplex images. Our approach uses a physically based stain model that simulates the light absorption of stains through the tissue. A channel selection strategy, using a lookup table or Large Language Model (LLM), allows for the mapping of molecular markers to their corresponding stain colors. To further enhance image quality, we integrate a deep learning-based upsampling and denoising model, trained on real brightfield images. We evaluated the methods on several modalities including mass-spectrometry based imaging mass cytometry and fluorescence based multiplex imaging. The results demonstrate that our method produces virtual brightfield images that are of similar quality as real brightfield images, are quantifiable and of diagnostic quality. We also show that LLMs are able to consistently determine appropriate channels in the multiplex image.

## Introduction

Recent advances in multiplex imaging allow us to visualize tissue architecture and protein expression at cellular resolution. This includes Imaging Mass Cytometry (IMC) (Giesen et al., 2014), which uses antibodies labeled with metal isotopes detected by laser ablation and mass spectrometry. Fluorescence-based methods include Cyclic Immunofluorescence (CycIF) (Lin et al., 2015), which performs iterative cycles of fluorescence imaging, and Co-detection by Indexing (CODEX) (Black et al., 2021), which employs DNA-barcoded antibodies. Multiplexing can also be extended to three dimensions using techniques such as Serial Two-Photon Tomography (STPT) (Ragan et al., 2012), which combines automated physical sectioning with two-photon microscopy, or by direct volumetric imaging of cleared tissues as with light-sheet microscopy.

Brightfield imaging using stains such as Hematoxylin and Eosin (H&E) complements multiplex imaging by incorporating clinical expertise (Lin et al., 2023). By deriving brightfield images from multiplexed data, the need for additional sample collection and complex double-staining methods is eliminated (Marlin et al., 2023), as is the requirement for aligning images when spatial correspondence is needed. Generating virtual or pseudo brightfield images from alternative modalities has also emerged as a valuable tool (Fanous et al., 2023). Virtual brightfield images provide a familiar visual context for pathologists, enhancing their ability to interpret tissue structures and inform diagnostic decisions. Moreover, by generating conventional color images, this approach eliminates the need for manual channel selection and adjustments of brightness and contrast in rendering multiplex images, allowing structures to be easily delineated and measured. Finally, these images enable the use of AI-based classification and segmentation tools that were trained on large datasets of real brightfield images.

There are two use cases where this method may be particularly useful. This is when the tissue sample is no longer available for subsequent staining, and when the tissue is scarce and no additional section is available for additional staining, such as with a needle biopsy. Some multiplex technologies allow H&E or other staining and brightfield imaging to be performed on the same section without loss of quality, which may require an additional registration step. However, this will typically allow only one additional stain to be applied. Generating virtual brightfield images from multiplex data, on the other hand, allows for any number of virtually stained images to be generated *post hoc*, depending on the markers included in the multiplex imaging.

Several studies have described methods and results on converting various modalities to virtual H&E images. These modalities include unstained histological tissue (Khan et al., 2023), label-free multispectral imaging Salido et al. (2023) and label-free autofluorescence imaging (Li et al., 2024; Wang et al., 2024). These methods have also been utilized to generate virtual representations of other histological stains (Yang et al., 2024; Rivenson et al., 2019). These methods, however, rely on deep learning to predict tissue structures to produce a virtual brightfield image, sometimes from a single channel image. The reliance on deep learning can lead to hallucinations, limiting their utility in clinical practice, quantitative research studies and other real-world applications. Furthermore, these models require modality-specific training data, restricting their generalizability to other imaging modalities or stain types.

Consequently, non-AI based methods remain more reliable and deterministic. Among these are simple additive methods (Gareau, 2009), as well as more advanced approaches that incorporate the physical properties of stains in light microscopy (Giacomelli et al., 2016; Simonson et al., 2021), which are applied in this study. A conceptual comparison with generative deep learning based approaches is provided in Table 1.

**TABLE 1:** Comparison between our proposed method and deep learning-based methods.

Property	Proposed	Deep learning-based
Image generation	Physics-based simulation	Learned
User control	High	Minimal
Determinism	Deterministic	Model-dependent
Interpretability	Explicit	Implicit
Risk of hallucination	Low	Non-negligible
Data fidelity	Explicit	Implicit
Generalizability	High across modalities and stains	Limited to training data

Our study presents a generalized software method for generating virtual brightfield images of any stain from any multiplexed imaging modality. By not relying on deep learning, our method allows for a deterministic simulation of any brightfield stain, while providing precise control over color and intensity parameters. We include the optional use of a Large Language Model (LLM) to select the appropriate markers for the colors in H&E or special stains. A deep learning based denoising and upsampling tool is provided to match clinical pathology image resolution and quality. This framework enables researchers and clinicians to create virtual brightfield images from any combination of molecular markers across different imaging modalities.

## Materials and methods

### Data

We used a number of datasets and tissue types to develop and qualitatively and quantitatively evaluate our algorithms. The datasets are listed below, with additional details provided in Table 2:

- Mouse-IMC-STPT: IMAXT (Imaging and Molecular Annotation of Xenografts and Tumors) IMC images of 2 mouse embryos with corresponding H&E images, and STPT data from one embryo. Data available at https://doi.org/10.6019/S-BIAD2843. All mouse experiments were performed under the Animals (Scientific Procedures) Act 1986 in accordance with UK Home Office licenses (Project License #PAD85403A) and approved by the Cancer Research UK (CRUK) Cambridge Institute Animal Welfare and Ethical Review Board.
- Human-Tonsil-IF-IMC: An Immunofluorescence (IF) image of tonsil tissue with corresponding H&E image and a closeup region imaged using IMC Marlin et al. (2023). Data available at https://doi.org/10.5281/zenodo.8023452.
- Human-Colorectal-Cancer-CyCIF: CyCIF images with registered H&E images Lin et al. (2023). Data download instructions at https://github.com/labsyspharm/orion-crc/blob/main/datarelease-README.md.
- Human-Breast-Cancer-IMC: IMC images of human breast cancer with cell annotations Jackson et al. (2019). Data available at https://doi.org/10.5281/zenodo.3518284.
- Human-SMIF: Sequential Multiplex IF unmixing (Akoya Vectra 3.0) images of various tissue types with cell annotations Aleynick et al. (2023). Data available at https://doi.org/10.7303/syn27624812.
- Human-Liver-Cancer-CODEX: CODEX images with clinical data Ruf et al. (2023). Data available at https://doi.org/10.7937/BH0R-Y074.

**TABLE 2:** Data table.

Dataset	Tech	Count	Markers	Resolution	Source	Tasks
Mouse-IMC-STPT	IMC	2	36	1 μ m	*de novo*	Illustrative
	STPT	30	3	0.56/25 μ ${\text{m}}^{a}$		
	H&E	2	-	0.5034 μ m		
Human-tonsil-IF-IMC	IF	1	4	0.3441 μ m	Public	Illustrative
	IMC	1	24	1 μ m		
	H&E	1	-	0.1721 μ m		
Human-colorectal-cancer-CyCIF	CyCIF	40	18	0.3250 μ m	Public	Quantitative comparison and patch prediction accuracy between real and virtual H&E
	H&E	40	-	0.3250 μ m		
Human-breast-cancer-IMC	IMC	735	35	1 μ m	Public	Enhancement efficiency And cell count accuracy
Human-SMIF	SMIF	130	8	0.4983 μ m	Public	Cell count accuracy
Human-liver-cancer-CODEX	CODEX	15	38	0.3774 μ m	Public	Pathologist evaluation

^*a*^0.56 μ m in-plane resolution, 25 μ m slice spacing.

### Stain calculation

Multiplex pathology imaging is typically displayed by assigning a different color to each channel and blending them together with a black background. However, simulating brightfield imaging requires a different approach since the background is bright and stains display color through light absorption.

A straightforward way to implement this is the additive (linear subtractive) method. Here, the channels in the multiplex image are assigned to predefined stain colors, and their respective contributions are simply summed and subtracted from the background color. This can provide reasonable approximations but does not realistically model the physics of light transmission.

Instead, we use a generalized version of a physics-based method (Giacomelli et al., 2016), which states that transmitted light decreases exponentially with the amount of absorbing material along the optical path. This produces more faithful representations of real brightfield histology imaging, since it mimics the nonlinear relationship between dye concentration and transmitted light.

To approximate brightfield imaging for digital microscopy and standard display devices, we simulate transmitted light at three representative wavelengths corresponding to the red (R) , green (G) , and blue (B) channels. As in the additive method, several stain classes can be defined, each with a color assigned to it. However, in the physics-based method, stain contributions are applied as an exponential attenuation of the background illumination in each RGB channel. Although initially proposed for virtual H&E, we generalize this method to any stain with an arbitrary number of markers and colors. Each stain class i is characterized by an RGB stain color $\left(S_{i,R},S_{i,G},S_{i,B}\right)$ , which determines the attenuation strength in channel c through $\left(c_{c}-S_{i,c}\right)$ , where c∈{R,G,B} .

In this model, the marker channel intensities are used to define the relative stain amount for each stain class, whereby the stain amount may be a combination of several markers. In addition, a scaling constant $k_{i}$ can be specified to control the overall staining strength of each stain class, which can be useful for adjusting the appearance to match reference brightfield images. Thus, for each stain class we first compute an intensity image $I_{i}$ from one or more marker channels, which is then used to compute the RGB output values as follows as detailed in Equations 1–3 below:

Intensity image:

$$\begin{array}{cc} & I_{i}=\frac{1}{N_{i}}\overset{N_{i}}{\underset{m=1}{\sum }}{\tilde{M}}_{i,m} \end{array}$$ (1)

Additive Method:

$$\begin{array}{cc} & \;R=c_{R}-\overset{n}{\underset{i=1}{\sum }}k_{i}I_{i}\left(c_{R}-S_{i,R}\right)\\ & G=c_{G}-\overset{n}{\underset{i=1}{\sum }}k_{i}I_{i}\left(c_{G}-S_{i,G}\right)\\ & \;B=c_{B}-\overset{n}{\underset{i=1}{\sum }}k_{i}I_{i}\left(c_{B}-S_{i,B}\right) \end{array}$$ (2)

Physical Method:

$$\begin{array}{cc} & \;R=d_{R}+\left(c_{R}-d_{R}\right)\exp\;\left(-\overset{n}{\underset{i=1}{\sum }}k_{i}I_{i}\left(c_{R}-S_{i,R}\right)\right)\\ & G=d_{G}+\left(c_{G}-d_{G}\right)\exp\;\left(-\overset{n}{\underset{i=1}{\sum }}k_{i}I_{i}\left(c_{G}-S_{i,G}\right)\right)\\ & \;B=d_{B}+\left(c_{B}-d_{B}\right)\exp\;\left(-\overset{n}{\underset{i=1}{\sum }}k_{i}I_{i}\left(c_{B}-S_{i,B}\right)\right) \end{array}$$ (3)

where:

-
${\tilde{M}}_{i,m}$ : normalized image of marker m assigned to stain class i

-
$N_{i}$ : number of markers assigned to stain class i

-
m : marker index (1 to $N_{i}$ )
-
$I_{i}$ : intensity image for class i

-
$S_{i,R}$ , $S_{i,G}$ , $S_{i,B}$ : reference color values for class i

-
$k_{i}$ : scaling constants for class i

-
$c_{R}$ , $c_{G}$ , $c_{B}$ : maximum (background) transmission values, all in range [0, 1]
-
$d_{R}$ , $d_{G}$ , $d_{B}$ : minimum transmission values, all in range [0, 1]
-
n : total number of classes
-
i : class index (1 to n )

**FIGURE 1:**
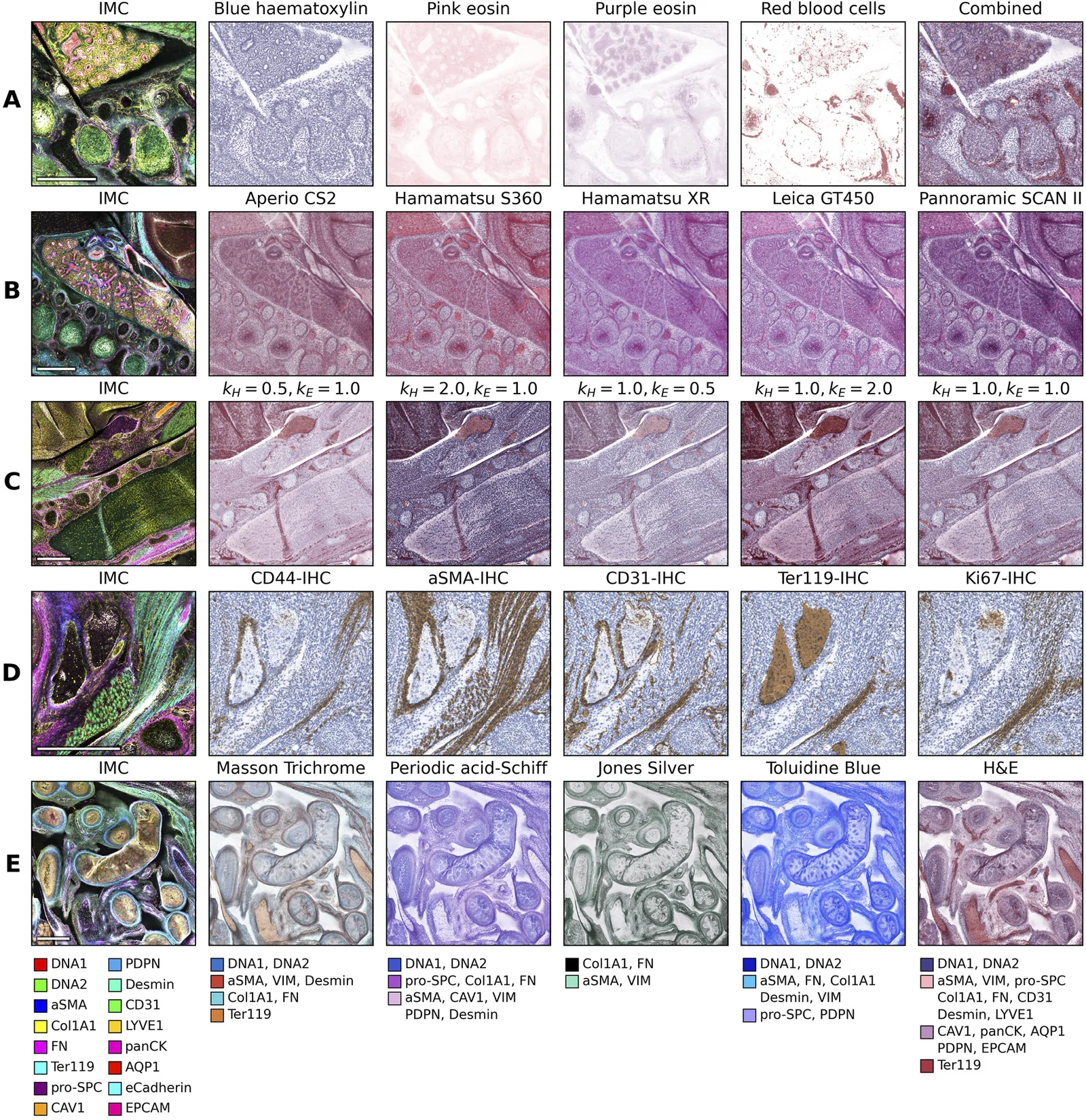
Composite images from Imaging Mass Cytometry data (Mouse-IMC-STPT) with corresponding virtual brightfield images. **(A)** The components of a virtual H&E image. **(B)** Color palette variations simulating different microscope systems. **(C)** The effect of varying intensity of hematoxylin $\left(k_{H}\right)$ and eosin $\left(k_{E}\right)$ . **(D)** Virtual Immunohistochemistry (IHC) images from different protein markers. **(E)** Special stains with the color classes and their corresponding protein marker channels. The scale bar indicates 500 μ m.

**FIGURE 2:**
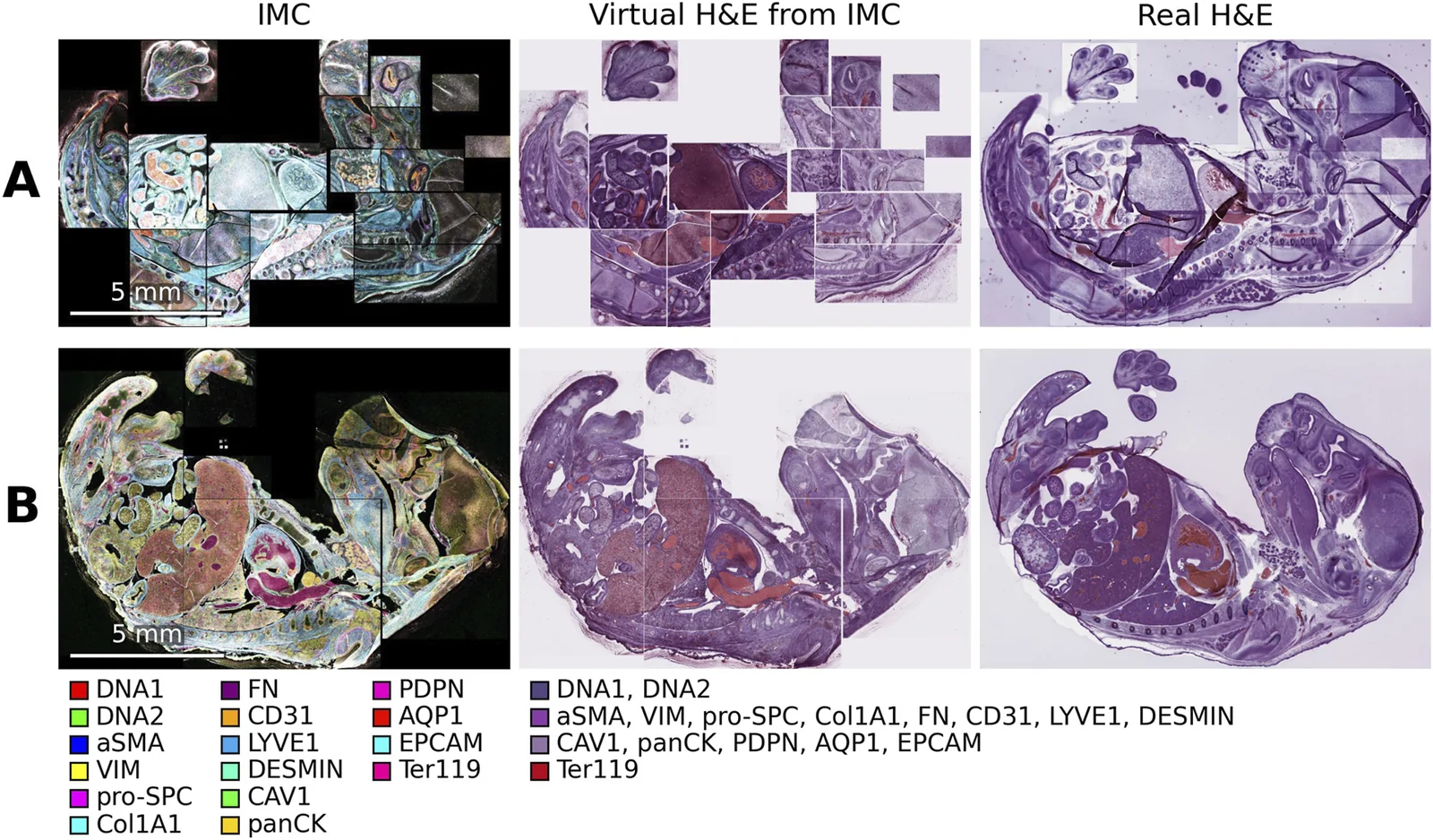
A side by side comparison of the virtual H&E generated from the IMC data and real H&E of 2 mouse embryos from the Mouse-IMC-STPT dataset, including the composite images of the IMC data with the markers used in generating the virtual H&E images. **(A)** In this sample the amount of ablation varied in the various regions resulting in some regions having higher or lower intensities in the composite IMC image and having more or less staining intensities in the virtual H&E images. **(B)** In this sample a different section of the same embryo was used for the real H&E.

The maximum transmission value represents the white background illumination, while the minimum transmission value represents the lowest detectable light of the microscope detector, which is typically black. In our implementation we allow for an arbitrary number of classes. For H&E for, instance, we define four classes, namely, blue hematoxylin (cell nuclei), pink eosin (extracellular matrix and cytoplasm), purple eosin (epithelial tissue), and red blood cells (erythrocytes) (Figure 1A). Each of which is assigned a stain color ( $S_{i,R}$ , $S_{i,G}$ , $S_{i,B}$ ). By adjusting these colors it is possible to have the color range appear similar to a real H&E image (Figure 2). We have defined default colors for the H&E stain for various microscopes (Figure 1B) by matching the colors with the microscope specific images from the Mitosis Domain Generalization Challenge (Aubreville et al., 2021, Aubreville et al., 2024). In addition, variations due to the staining, similarly to what was experimentally shown in Runz et al. (2021), can be set using the $k_{i}$ scaling constants (Figure 1C). For IHC, only a blue hematoxylin and brown color for the desired protein marker is defined (Figure 1D). Using this method we can also simulate any special stain by defining the colors resulting from the stain and selecting the channels that contribute to this color (Figure 1E).

**FIGURE 3:**
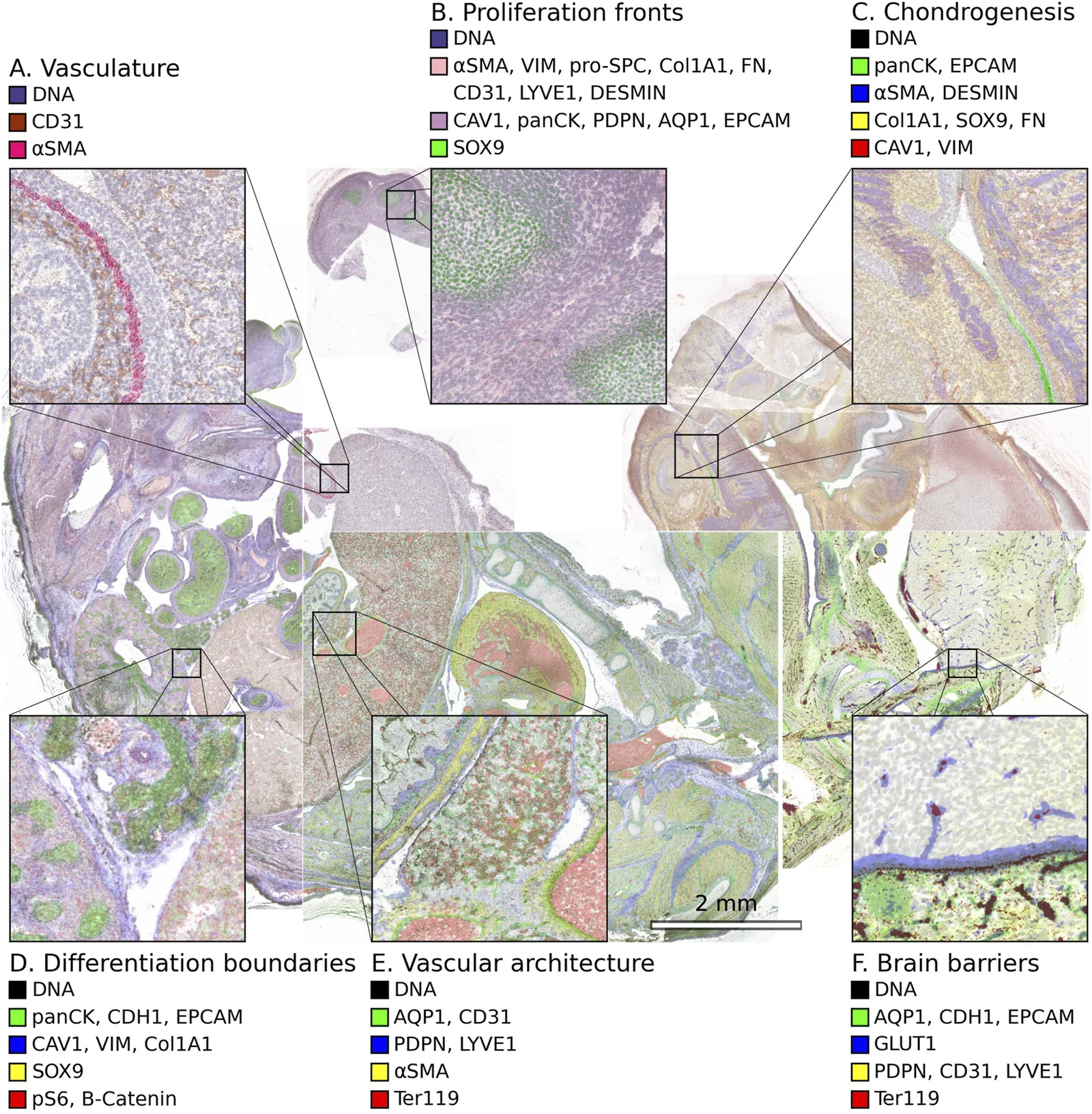
Examples of virtual brightfield with custom special stains within embryonic tissue regions (Mouse-IMC-STPT). **(A)** Dual-marker rendering of CD31 (brown) and α SMA (red), similar to conventional double IHC, used to highlight blood vessels and angiogenic features. **(B)** Virtual H&E image with an additional marker, SOX9, visualized in a contrasting color (green) to highlight proliferative progenitor niches. **(C)** A completely custom special stain combining cartilage progenitors (SOX9, yellow), mesenchyme (VIM/CAV1, red), epithelium (EPCAM/panCK, green), and muscle primordia (DESMIN/ α SMA, blue) to visualize craniofacial cartilage development. **(D)** An exploratory composite showing epithelial identity (EPCAM/panCK/CDH1, green), mesenchymal/ECM components (VIM/CAV1/Col1A1, blue), progenitors (SOX9, yellow), and signaling activity ( β -Catenin/pS6, red), useful for mapping epithelial-mesenchymal interfaces. **(E)** A custom special stain separating blood vessels (CD31 ± AQP1, green), lymphatic endothelium (PDPN/LYVE1, blue), vascular smooth muscle ( α SMA, yellow), and erythrocytes (Ter119, red), providing a single-view map of vessel types and wall structure. **(F)** A custom special stain combining blood-brain barrier (GLUT1, blue), choroid plexus epithelium (AQP1/CDH1/EPCAM, magenta), meningeal lymphatics (PDPN/LYVE1/CD31, green), and erythrocytes (Ter119, red), to visualize distinct vascular and barrier systems in the brain.

In addition to simulating H&E, IHC, or common special stains, our methodology can be extended to generate stains that are difficult or even impossible to achieve with multiplexed immunohistochemistry (mIHC). Several examples are shown in Figure 3 in different regions of a mouse embryo imaged by IMC. These include virtual IHC with DAPI in blue, vasculature and lymphatic vessels in brown, and GLUT1 in red (Figure 3A), similar to what can be obtained in conventional double-stained IHC. The method also demonstrates how a bright contrasting green can be combined with standard H&E, in this case to highlight proliferation fronts using SOX9 (Figure 3B). More broadly, this approach enables the rendering of any cellular, tissue, or functional markers in any chosen color, allowing more elaborate evaluation of tissue samples and a direct visualization of the relationships between different tissue types (Figures 3C–F). The composite IMC image and the virtual H&E image of this section, as well as a real H&E image of a different section of this embryo are provided in Figure 2B.

**FIGURE 4:**
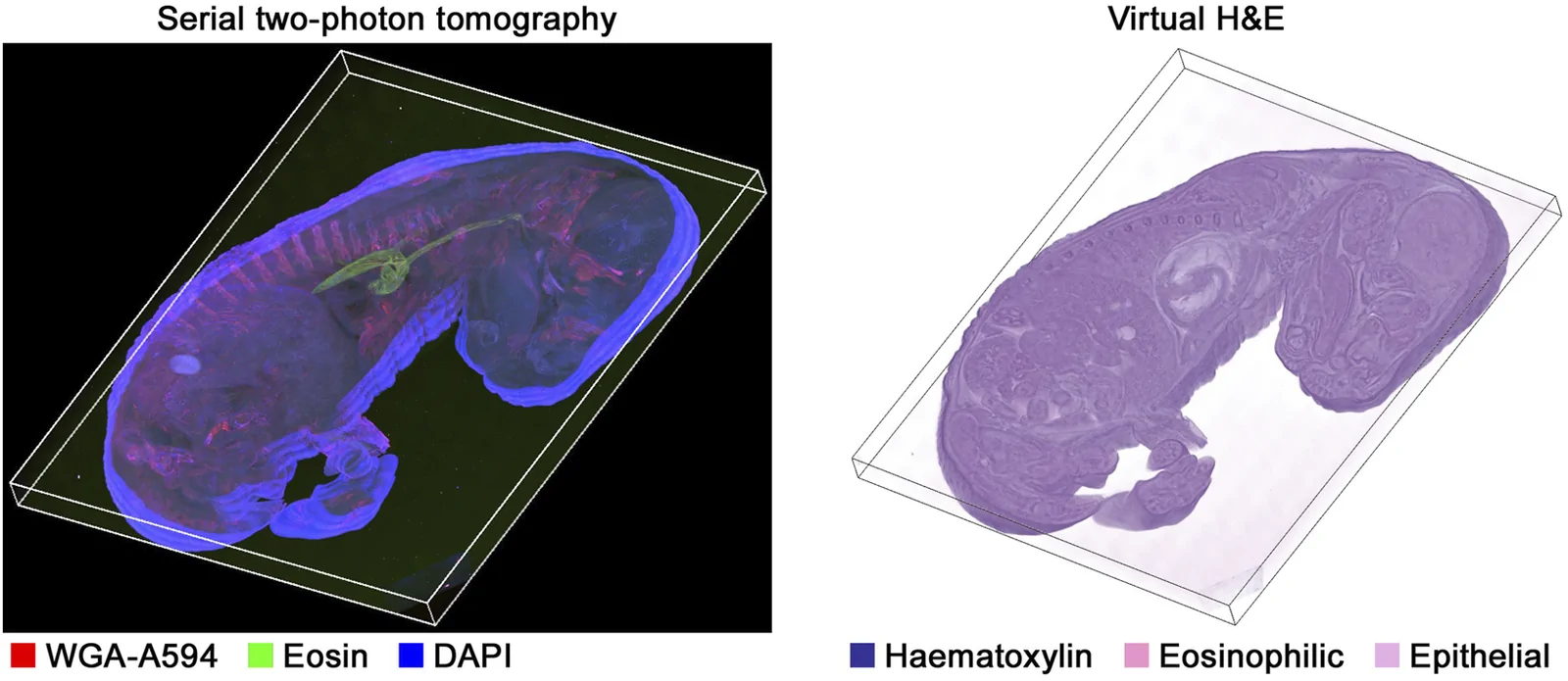
3D virtual H&E from 30 sections at a 25 μ m slice thickness of serial two-photon tomography data (Mouse-IMC-STPT). WGA-A594 refers to wheat germ agglutinin conjugated to Alexa Fluor 594, a fluorescent lectin that labels cell membranes. Additive rendering of three channels from the STPT data (left) visualized using cyto-studio, and the volume rendering of the virtual H&E color volume (right) generated using ScanXm (Minogame Limited, Cambridge, United Kingdom).

All of these methods can also be applied to generate 3D brightfield volumes from 3D immunofluorescence data. An example is shown in Figure 4 for a mouse embryo, where an H&E volume was generated from STPT (Mouse-IMC-STPT) data, which would otherwise require spatial alignment of serial histological sections. A video of this volume rendering is provided in Supplementary Data 1.

### Channel selection

Selecting the appropriate markers to represent colors in the virtual brightfield image can be straightforward when highly specific markers are available. For example, when generating H&E images, one might use DAPI for blue nuclear staining, collagen or fibronectin markers for the pink extracellular matrix, and cytokeratin, vimentin, or Caveolin-1 for producing a purple hue in epithelial or other dense structures. Red blood cells can be targeted using hemoglobin markers.

In cases where specific markers for the desired stain are unavailable, channels can be grouped into predefined stain classes. For instance, the pink class in H&E may include all structural proteins predominantly located in the cytoplasm and extracellular matrix. Although the user can manually specify the markers for each stain class, for several stains we provide configuration presets that define stain classes with a default color and a curated list of associated marker names (including common aliases and naming variations). During processing, the channel names in the input multiplex image are screened against the predefined marker lists corresponding to the selected stain. Matching channels are then grouped into the appropriate stain class, while irrelevant channels are ignored. The intensity image $I_{i}$ for each class i is then computed by averaging the images of the assigned channels.

### LLM-based channel selection

While such a predefined marker list suffices in most cases, it will not cover some unconventional special stains, and may not work in multiplex data with unconventional channel naming conventions. To address this, we offer an optional LLM-based approach to automatically select the appropriate channels through an API call to ChatGPT (OpenAI, San Francisco, United States), Gemini (Google DeepMind, London, UK) or Claude (Anthropic, San Francisco, United States). The LLM is provided with a prompt that explains the task at hand, the name of the stain, and its associated classes with a description and color, as well as the complete list of channel names from the multiplex image. The output of the LLM is then parsed to obtain the list of channels for each class, which are then used in the subsequent image synthesis process.

### Post processing

**FIGURE 5:**
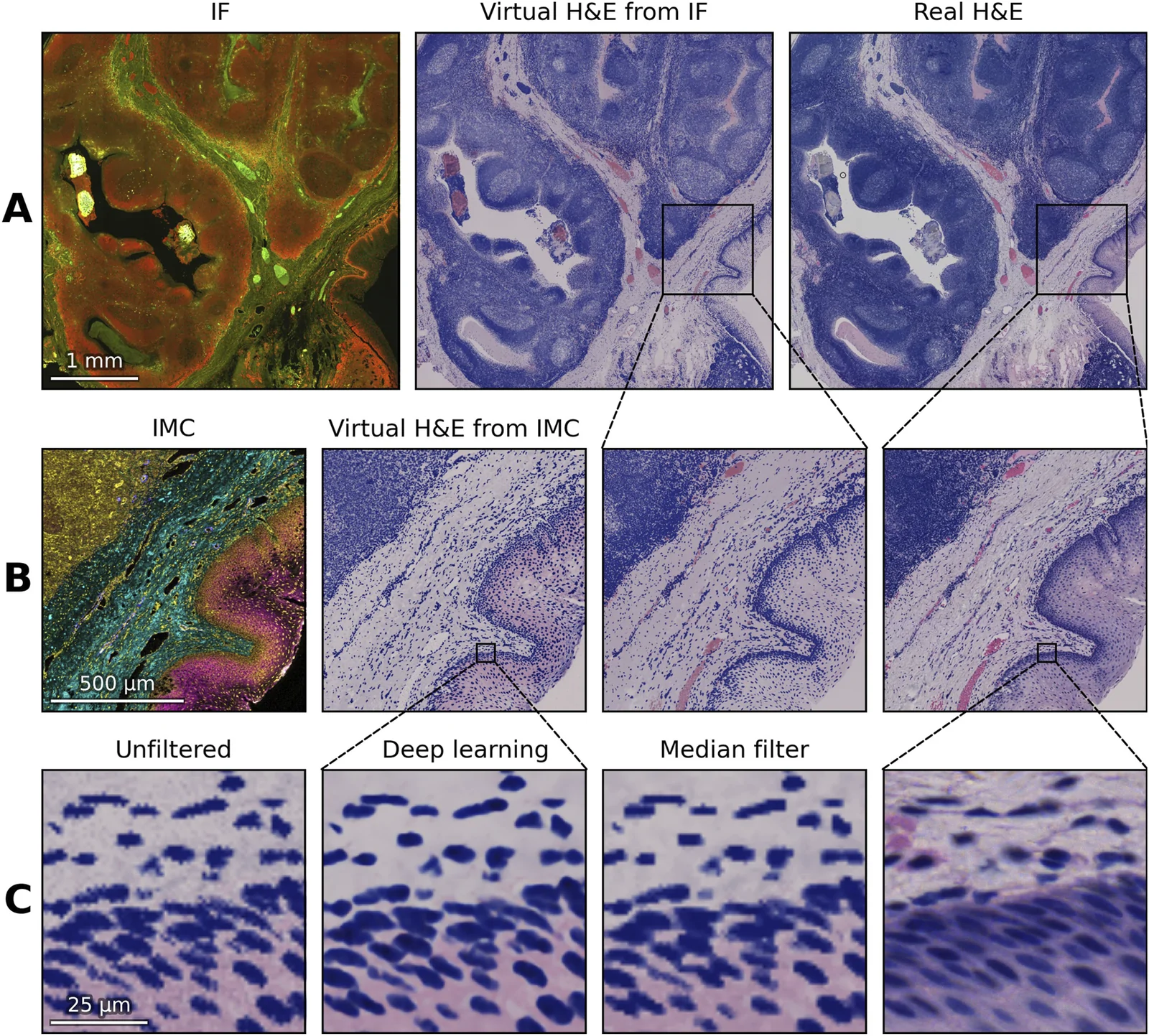
Comparison of virtual H&E images generated from an immunofluorescence (IF) and Imaging Mass Cytometry (IMC) image of tonsil tissue from the Human-Tonsil-IF-IMC dataset with corresponding real H&E images, all of the same section. **(A)** Multiplex immunofluorescence composite image of the tonsil tissue, showing nuclear and multiple protein marker channels, together with the corresponding virtual H&E image and the real H&E image. Adaptive histogram normalization was applied to the nuclear channel in the virtual H&E image. **(B)** IMC composite image of a subsection, representing multiple metal-tagged protein markers, shown alongside its virtual H&E image. The same cropped region of the virtual H&E image from IF and the real H&E image are provided as comparison. **(C)** Three versions of a closeup region of the virtual H&E from IMC, upscaled from 1 μ m to match the 0.1721 μ m resolution of the real H&E image. From left to right: the image unfiltered and B-spline upscaled, the image upscaled using nearest neighbor interpolation and filtered using the deep learning-based method, the image filtered with a median filter and then B-spline upscaled and the approximate same region in the real H&E image.

A number of optional post-processing tools are included to enhance the realism of the resulting virtual brightfield image (Figure 5). These include a median filter to remove the noise, such as in IMC images, or a sharpening filter to de-blur the signal in fluorescence imaging. It also includes adaptive histogram normalization which, when applied to the hematoxylin channel, ensures that all cells have the same intensity range throughout the tissue. The image can also be up-scaled with a B-spline based resampling if the resolution of the multiplexed image is smaller than the resolution of a standard brightfield image, or downscaled otherwise.

### Deep learning based post processing

We also include a more advanced post-processing tool based on deep learning. For this we adopted NAFNet (Chen et al., 2022), originally developed for image restoration. In contrast to generative approaches such as Generative Adversarial Networks (GANs) like pix2pix (Tahmid et al., 2023) or diffusion models (Ho et al., 2020), which can prioritize photorealism at the expense of faithfulness, NAFNet functions as a deterministic restoration network, minimizing the risk of hallucinations. To reinforce this objective during training, we employed a composite loss comprising an L1 term, a structural similarity (SSIM) term, and an additional image-gradient loss. The L1 term constrains the output to remain faithful to the reference, the SSIM term counteracts oversmoothing, and the gradient term explicitly penalizes discrepancies in edge gradients, thereby encouraging preservation of fine local structures such as nuclei and tissue boundaries.

The network was reimplemented in TensorFlow and Keras with minor adaptations, including the use of strided convolutions and nearest-neighbor upsampling instead of pixel shuffle and unshuffle.

To have the model function as a super-resolution upscaler, during training, the input image is randomly down-scaled and then up-scaled back to the original size using a nearest neighbor interpolation. This introduces stair-step artifacts, which give the model clues into the original resolution, allowing it to appropriately enhance the image. The input is also randomly blurred, encouraging the network to perform deblurring during inference.

At the same time, we also train the model to remove noise by adding simulated noise to the input image. Given the various possible multiplex imaging modalities, this noise may include Gaussian noise, but also Poisson noise, and may include hot pixels. The noise is added in a conventional way to the RGB components, or to the components in the HSV color space. However, to more accurately simulate noise for IMC or fluorescence-derived virtual H&E images, we also decompose the image into a haematoxylin, eosin and DAB components, apply Gaussian, Poisson or hot pixel noise to each component, and then convert the image back to RGB color space. Additionally, standard color jitter and random rotations and flips are applied.

When post-processing the virtual brightfield images with this model, they are first rescaled to the desired resolution using nearest neighbor interpolation, after which the model is applied via a sliding window approach.

### Training data

A data set of H&E and various IHC images at 20 and 40 times magnification were collected for the training of the denoising and up-sampling model. Of the Cancer Imaging Archive we used image regions from icdc-glioma Amin et al. (2020), her2-tumor-rois Farahmand et al. (2022), cptac-brca National Cancer Institute Clinical Proteomic Tumor Analysis Consortium (2020), cptac-hnscc National Cancer Institute Clinical Proteomic Tumor Analysis Consortium (2018c), cptac-ccrcc National Cancer Institute Clinical Proteomic Tumor Analysis Consortium (2018a), ptrc-hgsoc Chowdhury et al. (2023), cptac-cm National Cancer Institute Clinical Proteomic Tumor Analysis Consortium (2018b), cptac-sar National Cancer Institute Clinical Proteomic Tumor Analysis Consortium (2019) and cmb-mel Cancer Moonshot Biobank (2022).

We also included the images from the PanNuke Dataset Gamper et al. (2019), the 2021 and 2022 Mitosis Domain Generalization Challenge (Aubreville et al., 2021, Aubreville et al., 2024), and, to support various IHC images, we included images from nadt-prostate (Wilkinson et al., 2020).

To add robustness to other stains we also included the images from histopathology textbooks and articles of the ARCH dataset Gamper and Rajpoot (2021) (available at https://warwick.ac.uk/fac/cross_fac/tia/data/arch/).

## Results

As recommended in Bai et al. (2023), we performed quantitative image-quality analyses, algorithm-based quantitative feature analyses, and a pathologist-assisted evaluation.

For the quantitative metrics, we compared virtual H&E images from the Human-Colorectal-Cancer-CyCIF datasets with their associated registered H&E images. Because the full slide images are too large for direct computation, we divided each image into regions of 8192 × 8192 pixels and averaged the results over all regions for each sample. This resulted in the following measures reported as mean ± standard deviation. The pixel-level agreement was quantified using the Pearson Correlation Coefficient (PCC, 0.62 ± 0.07) and Mean Squared Error (MSE, 807 ± 811). The structural and textural fidelity was evaluated with the Multi-Scale Structural Similarity Index Measure (MS-SSIM, 0.72 ± 0.06), and the color distribution accuracy was assessed using the Earth Mover’s Distance (EMD, 15.7 ± 12.3) alongside the Peak Signal-to-Noise Ratio (PSNR, 20.8 ± 2.5) for pixel-wise reconstruction accuracy.

**FIGURE 6:**
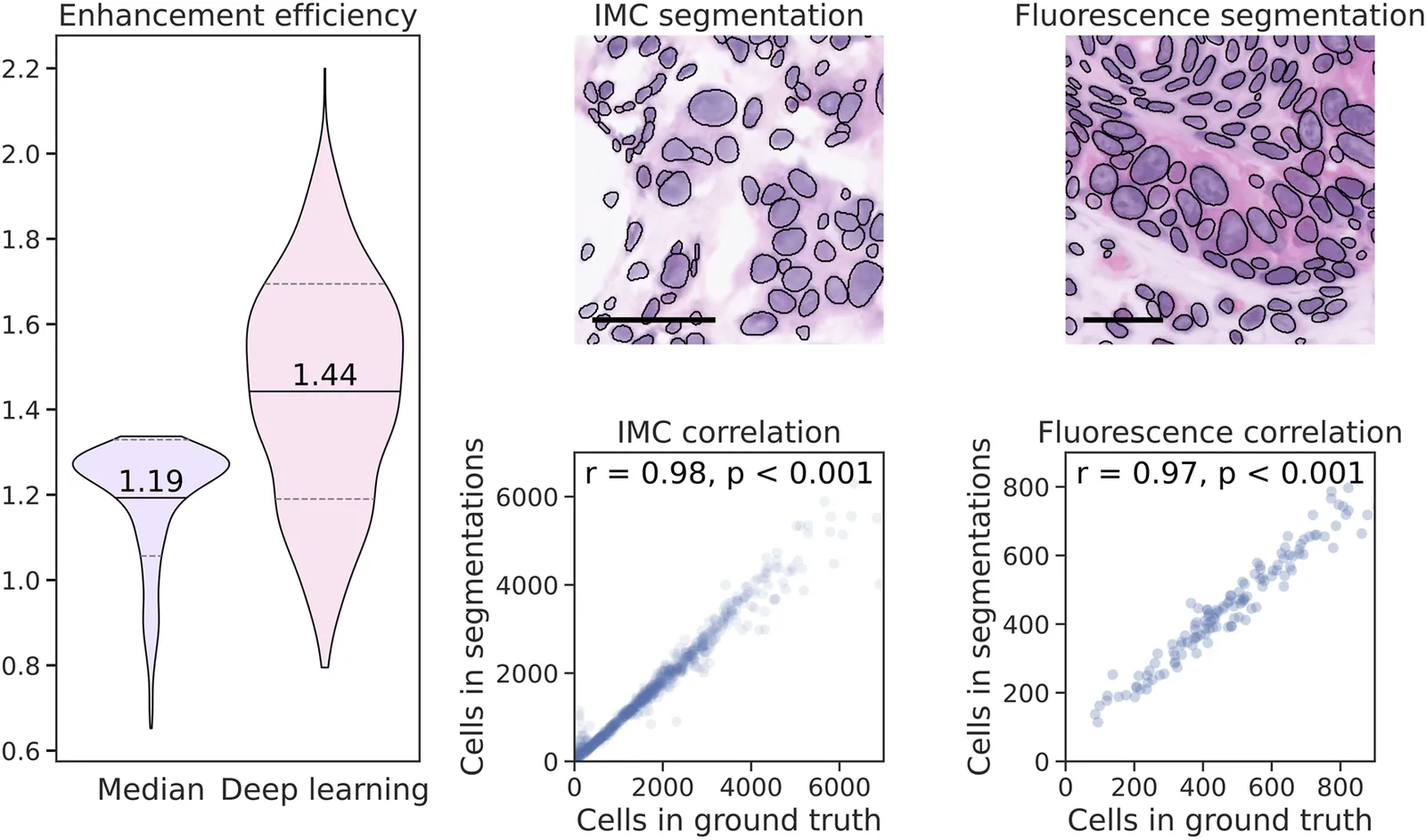
Left: Violin plots of the enhancement efficiency index for median-filtered and B-spline-upscaled (Median) and deep learning-enhanced (Deep learning) virtual H&E images from IMC data (Human-Breast-Cancer-IMC), upscaled from 1 μ m to 0.5 μ m pixel size. The solid line and values indicate the mean, and the dashed lines indicate the standard deviation. Right-Top: Representative samples from the Human-Breast-Cancer-IMC (IMC) and Human-SMIF (Fluorescence) datasets with StarDist cell segmentations shown as black contours. Right-Bottom: Correlation plots of cell counts in the virtual H&E images of both datasets compared with their respective ground truth references, both processed using the deep learning-based enhancement at 0.5 μ m pixel size.

We evaluated the efficacy of the deep learning-based upsampling and denoising model using the Human-Breast-Cancer-IMC dataset, with the virtual H&E images upsampled from 1 μ m to a clinically relevant 0.5 μ m resolution. Since aligned real H&E reference images were not available, we introduced an enhancement efficiency index (E) to quantify the trade-off between structural preservation and noise suppression, normalized to the corresponding raw image. The index is defined as in Equation 4:

$$\begin{array}{cc} & E=\frac{\left(edge_{f}/edge_{u}\right)}{\left(noise_{f}/noise_{u}\right)} \end{array}$$ (4)

where edge denotes the mean Sobel gradient magnitude and noise represents the standard deviation of high-frequency residuals. Subscripts f and u correspond to the filtered and unfiltered images, respectively. Using this metric, we compared a conventional median filter with the deep learning method. The results show that the deep learning-based approach achieves a higher enhancement efficiency, indicating a more favorable balance between noise reduction and edge preservation (Figure 6).

For the algorithm-based quantitative feature analysis, we calculated the number of cells in the virtual H&E images from the Human-Breast-Cancer-IMC and Human-SMIF datasets using the StarDist cell segmentation tool Schmidt et al. (2018) and compared the results with the ground truth via correlation analysis, indicating very strong correlations (Figure 6).

**FIGURE 7:**
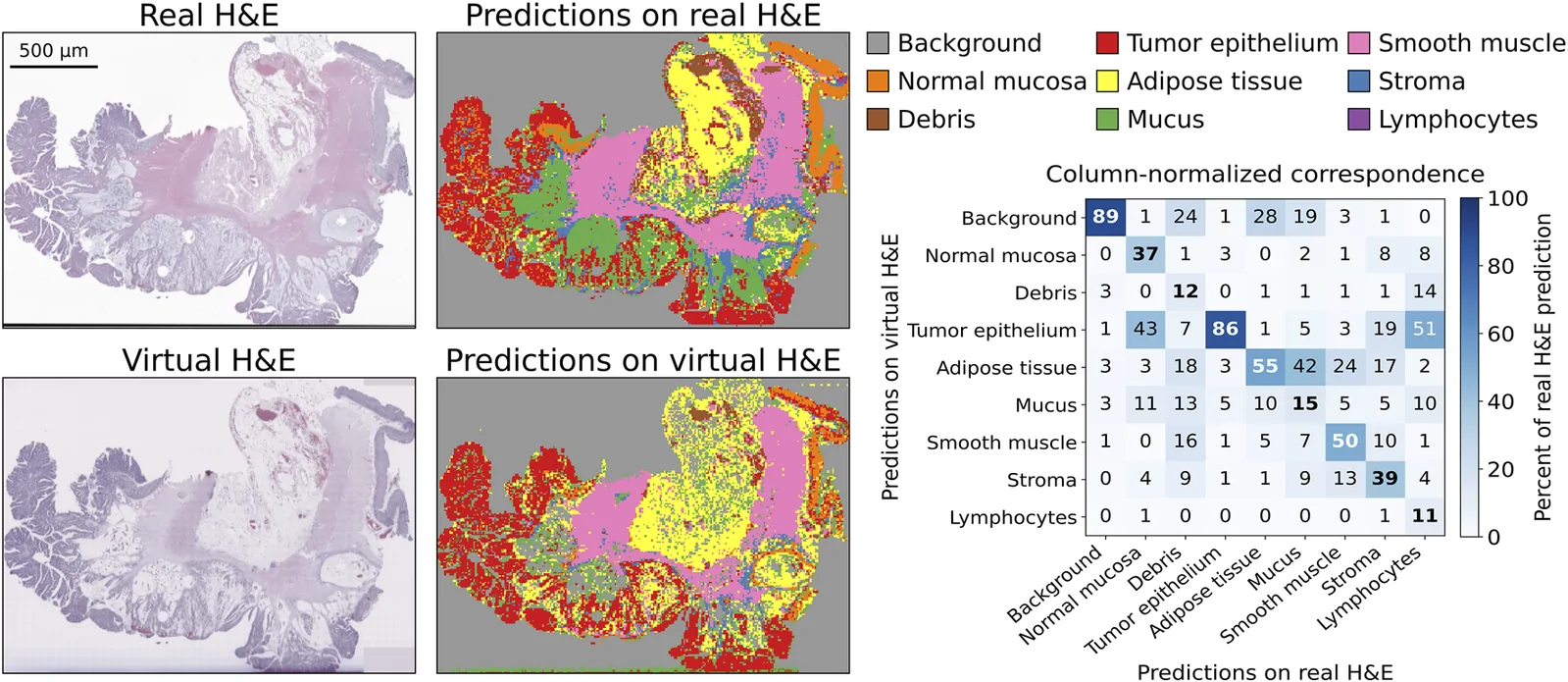
Example real and virtual H&E images from Cyclic Immunofluorescence (CyCIF) data of the Human Colorectal Cancer CyCIF dataset, with model predictions and the aggregate tile-level correspondence. On the left, a virtual H&E image is shown with its registered real H&E counterpart, together with tissue-type predictions generated using the TIAToolbox patch-based pipeline. The heatmap on the right summarizes results across all paired slides as a column-normalized confusion matrix. Diagonal cells indicate the percentage of correctly identified tiles for each real tissue class, while off-diagonal entries highlight systematic confusions.

We also employed the TIAToolbox for patch-based prediction using the ResNet34-Kather100k model, trained on the NCT-CRC-HE-100K dataset Kather et al. (2018), which contains patches of human colorectal cancer and healthy tissue with associated labels. We applied this patch-based predictor to the virtual H&E images generated from CyCIF data of the Human-Colorectal-Cancer-CyCIF dataset, as well as to the corresponding real, registered H&E images. This allowed us to compare prediction correspondence between real and virtual H&E, providing a measure of how well the virtual images mimic tissue type based on this pretrained histology classifier (Figure 7).

For the pathologist-assisted evaluation, 15 human liver cancer samples with hepatocellular carcinoma were assessed using the virtual H&E images generated from the Human-Liver-Cancer-CODEX dataset. Reference tumor grades (three-tier histopathological grading) were obtained from the clinical metadata provided with the Human-Liver-Cancer-CODEX dataset on The Cancer Imaging Archive (TCIA). A pathologist blinded to the reference labels graded each virtual H&E image. Agreement with the reference grades resulted in an overall accuracy of 80% and a quadratic weighted Cohen’s κ of 0.84.

Finally, the ability of an LLM to accurately select the appropriate channels associated with each color/class was assessed using the 36 channel names in the Mouse-IMC-STPT dataset. Because no ground truth annotations were available, we determined a consensus from *ChatGPT 5 (Thinking Extended)*, *Gemini 2.5 Pro* and *Claude Sonnet 4.5*, which were prompted to assign channels to the color classes of several special stains. The full prompts are provided in the code release. For each stain, we calculated the consensus Jaccard index, which we defined as the mean Jaccard similarity between each model and the consensus across all classes, and then averaged these values across the three models (Table 3). The results show that the LLMs achieve consistently high overlap across all stains, highlighting their potential for reliable and automated marker assignment in multiplexed imaging.

**TABLE 3:** Mean consensus Jaccard index showing the correspondence between the three LLMs (*ChatGPT 5 (Thinking Extended)*, *Gemini 2.5 Pro*, and *Claude Sonnet 4.5*) in selecting the channels of a multiplexed image for the classes of various stains in the Mouse-IMC-STPT dataset.

Stain	The classes with distinct colors	Jaccard index
Masson trichrome	Nuclei, muscle, collagen, erythrocytes	0.903
Periodic acid-Schiff	Nuclei, polysaccharides, stroma	0.719
Jones silver	Membranes, stroma	0.822
Toluidine blue	Nuclei, stroma, metachromasia	0.706
H&E	Nuclei, eosinophilic, epithelial, erythrocytes	0.909

## Discussion

Multiplex imaging technologies such as immunofluorescence, imaging mass cytometry, and CODEX provide highly detailed molecular information but are often difficult to interpret. In contrast, brightfield imaging with H&E or IHC remains the clinical standard because of its familiarity, visual clarity, and extensive diagnostic knowledge base. In this study, we introduce a toolkit that converts multiplexed data into virtual brightfield images, allowing researchers and pathologists to explore complex multiplex datasets in a format that closely resembles conventional stains.

Conventional double IHC typically combines horseradish peroxidase with DAB (3,3′-diaminobenzidine), the standard brown chromogen used in IHC, and alkaline phosphatase with a red chromogen such as Permanent Red, allowing two antigens to be visualized simultaneously in a single section. Recent advances in multiplex immunohistochemistry (mIHC) have shown that brightfield imaging can be extended beyond single-marker chromogenic stains to detect multiple biomarkers on the same tissue. Our framework can generate similar images from any multiplex imaging modality and further extends this capability by enabling virtual stains that are not feasible with current chromogenic methods (Figure 3), thereby enhancing tissue interpretability.

Our results demonstrate that the generated virtual brightfield images closely approximate the appearance of real H&E and IHC, both in terms of color fidelity and structural detail. Quantitative comparisons with registered H&E images showed strong correlations for pixel-level agreement, texture, and color distribution. Cell counts derived from real and corresponding virtual H&E images also showed strong correlation for both IMC and fluorescence imaging.

Tissue classification showed good correspondence in identifying tumor tissue, with 86% correctly classified relative to predictions from real H&E images (Figure 7). Some tissues that were not correctly identified, such as mucus, can be explained by the absence of relevant markers, whereas other discrepancies, such as those involving lymphocytes, may result from differences between the appearance of the training and test data. While the DNA marker provides a robust signal to simulate the hematoxylin nuclear stain, the eosin component is approximated from a combination of available protein channels rather than from a dedicated cytoplasmic stain. As a result, some features that are important for tissue type classification may not appear the same as in real H&E. Some color differences may also arise since the stain colors and scaling parameters are manually tuned to approximate the H&E images of this dataset. Since the classifier was trained on conventional brightfield images, any differences in color distribution and texture between real and virtual H&E can lead to unexpected behavior, such as a confusion between lymphocyte and tumor epithelium.

Importantly, a pathologist blinded to the clinical data was able to assess tumor grade from virtual H&E images with high concordance to real slides, supporting the diagnostic quality of the method. Overall, these findings indicate that virtual brightfield images derived from multiplex data are not only visually convincing but also preserve the quantitative and diagnostic properties required for downstream pathology workflows.

The deep learning based upsampling and denoising can be seen to result in higher quality images (Figure 5), but also quantitatively are shown to be of greater efficiency in enhancing the images compared to a traditional median filter (Figure 6). Although the model is designed to mitigate hallucinations, there remains a possibility that the model could introduce false structures, such as misinterpreting clusters of hotspots as cells, in particularly when the resolution is small and the deep learning model attempts to upsample a lot. If the pixel size is not large enough to distinguish cells (approximately >1 um) the images should be examined for apparent hallucinations. For cases where such concerns arise, conventional filtering methods are also available as an alternative. Also the use of the LLM for determining the markers used for the various stain colors should be used with care. Especially with ambiguous channel naming, unconventional stains and when used in a downstream analysis. In this case the user should always verify that the markers selected by the LLM are appropriate.

Naturally, this method will not replace regular H&E or IHC in most use cases, as multiplex imaging remains costly, complex, and less widely available. Nonetheless, it can add value in specific scenarios, such as a retrospective setting where the tissue is no longer available for additional staining, or when limited tissue prevents acquiring an additional section for conventional staining. In research, virtual brightfield views of multiplex data enable direct comparison of molecular marker distributions with tissue morphology in a familiar format, reducing the need for separate staining. This is particularly useful in spatial biology, where functional markers such as proliferation, hypoxia, or immune checkpoint proteins must be interpreted in their structural context. More broadly, the approach offers convenience and familiarity by displaying multiplex data without additional sections or staining, while preserving interpretability within the framework of brightfield pathology.

Several challenges remain. For large datasets, the computational load can be considerable. The computation time ranges from under 1 s for 400 × 400 pixels with 8 channels (Human-SMIF dataset) to approximately 3 hourd for 78,417 × 57,360 pixels with 26 markers (Human-Liver-Cancer-CODEX dataset). Additional multi-threading may improve performance. While default parameter values generally provide a reasonable starting point, further tuning may be required to achieve optimal results, which can be time consuming. Automated tools to sample parameters on smaller regions could help accelerate this process by suggesting appropriate settings and allowing rapid previewing.

At present, the library supports only the OME-TIFF format. To address this, we provide converters from CZI to OME-TIFF (via the *czi2ometiff* package)^1^ and from NumPy arrays to OME-TIFF (via the *numpy2ometiff* package)^2^. Future development will focus on adding direct support for widely used formats such as DICOM-WSI, Zarr, and SVS. Community contributions through GitHub are encouraged to help address these challenges and expand the utility of the toolkit.

To conclude, we presented a robust framework for generating virtual brightfield images from multiplexed imaging. By combining a physically based stain model, automated channel selection via large language models, and deep-learning-based denoising and upsampling, our method produces diagnostically relevant images. Quantitative and qualitative evaluations confirm its accuracy and utility, highlighting its potential to enhance histopathological analysis from multiplexed imaging.

## Data Availability

The datasets generated in this study (Mouse-IMC-STPT) are publicly available from the BioImage Archive under DOI: https://doi.org/10.6019/S-BIAD2843. Previously published datasets used for benchmarking, validation, and model training are publicly available from their respective repositories and are referenced in the Materials and Methods section. The source code for the multiplex2brightfield software is openly available at https://github.com/TristanWhitmarsh/multiplex2brightfield. The analysis code used to generate the figures is available at https://github.com/TristanWhitmarsh/multiplex2brightfield-analysis.

## Footnotes

1 Available on PyPI: pip install czi2ometiff.

2 Available on PyPI: pip install numpy2ometiff.
